# *GmHXK2* promotes the salt tolerance of soybean seedlings by mediating AsA synthesis, and auxin synthesis and distribution

**DOI:** 10.1186/s12870-024-05301-3

**Published:** 2024-06-27

**Authors:** Yuqi Guo, Chang Liu, Shuai Chen, Zengyuan Tian

**Affiliations:** 1 School of Life Sciences, Zhengzhou University, Zhengzhou, Henan PR China; 2 School of Agricultural Sciences, Zhengzhou University, Zhengzhou, Henan PR China

**Keywords:** GmHXK2, Salt stress, Lateral root, Auxin, AsA

## Abstract

**Background:**

Salt is an important factor that affects crop productivity. Plant hexokinases (HXKs) are key enzymes in the glycolytic pathway and sugar signaling transduction pathways of plants. In previous studies, we identified and confirmed the roles of *GmHXK2* in salt tolerance.

**Results:**

In this study, we analyzed the tissue-specific expression of *GmHXK2* at different growth stages throughout the plant’s life cycle. The results showed that *GmHXK2* was expressed significantly in all tissues at vegetative stages, including germination and seedling. However, no expression was detected in the pods, and there was little expression in flowers during the later mature period. Arabidopsis plants overexpressing the *GmHXK2* (OE) had more lateral roots. The OE seedlings also produced higher levels of auxin and ascorbic acid (AsA). Additionally, the expression levels of genes *PMM, YUC4/YUC6/YUC8*, and *PIN/LAX1,LAX3*, which are involved respectively in the synthesis of AsA and auxin, as well as polar auxin transport, were upregulated in OE plants. This upregulation occurred specifically under exogenous glucose treatment. *AtHKT1*, *AtSOS1*, and *AtNHX1* were up-regulated in OE plants under salt stress, suggesting that *GmHXK2* may modulate salt tolerance by maintaining ion balance within the cells and alleviating damage caused by salt stress. Additionally, we further confirmed the interaction between GmHXK2 and the protein GmPMM through yeast two-hybridization and bimolecular fluorescence complementation assays, respectively.

**Conclusion:**

The expression of *GmHXK2* gene in plants is organ-specific and developmental stage specific. *GmHXK2* not only regulates the synthesis of AsA and the synthesis and distribution of auxin, but also promotes root elongation and induces lateral root formation, potentially enhancing soil water absorption. This study reveals the crosstalk between sugar signaling and hormone signaling in plants, where GmHXK2 acts as a glucose sensor through its interaction with GmPMM, and sheds light on the molecular mechanism by which *GmHXK2* gene is involved in salt tolerance in plants.

**Supplementary Information:**

The online version contains supplementary material available at 10.1186/s12870-024-05301-3.

## Background

Glucose serves as energy for various life activities in plant cells. Additionally, it plays a crucial role in resisting biotic and abiotic stress, acting as a signaling molecule during the entire development stage of plants [1, 2]. Glucose can interact with plant hormones such as cytokinins [3] gibberellins [4], abscisic acid [5], ethylene [6, 7], brassinosteroids [8], and other signaling molecules, and participates in various signal transduction through comprehensive crosstalk [9, 10].

The pathways triggered by glucose signals, both hexokinase-dependent and non-hexokinase-dependent, mediate plant physiological reaction and development process [11, 12]. The plant hexokinase was firstly discovered in *Triticum aestivum L.* [13]. In 1997, Jang [14] confirmed the existence of hexokinase isoenzymes in plants, and then hexokinase genes family have been identified in various plants, such as Arabidopsis [15], rice [16], maize [17], cotton [18], tomato [19], and so on. Hexokinase not only transfers phosphate group to hexose in plants, but also plays an important role in regulating plant development under stress conditions as a glucose sensor. *AtHXK1* coordinates light and endogenous glucose signals to regulate plant development in signal pathways [4, 12]. It can also enhance plant tolerance to drought and salt stress [20, 21]. Karve confirmed that *AtHKL1* mediates the crosstalk between sugar and some plant hormone signaling pathways, negatively regulating plant growth [22]. The apple MdHXK1 participates in the plant’s salt stress process by interacting with MdNHX1 [23]. Inhibition of expression in *OsHXK10* leads to a failure of anther dehiscence and a decrease in pollen germination rate [16].

Auxin plays a critical role in promoting growth, regulating the cell cycle, and responding to environmental stimuli in various stages of the plant lifecycle [9, 10]. During cell division and plant growth, auxin can be effectively transported from the synthesis site to the target site [24]. The distribution of auxin is performed by intracellular carriers, which include auxin influx carriers AUX/LAX [25], auxin efflux carriers PIN-FORMED (PIN), and p-glycoproteins [26, 27]. Auxin activates or inhibits the expression of auxin response genes through auxin response factors (ARFs) and AUX/IAA inhibitory factors. ARFs specifically bind to the TGTCTC auxin-responsive elements (AuxRE) in the promoter of auxin response genes, thus promoting the expression of target genes [27–29]. The content and distribution of endogenous auxin in plants can be analyzed by a highly active synthetic auxin element DR5 [30] as the promoter to drive the expression of the GUS gene. Sugar can cooperate with auxin signals to regulate plant growth, development, and morphology [9]. The combined effect of both signals on growth in plants was first studied in Arabidopsis, where the *gin2* mutant displayed insensitivity to sugar and resistance to exogenous auxin [31]. Mutants with auxin signaling defects were insensitive to high concentrations of exogenous glucose. Additionally, mutants of the HLS1 gene, an acetyltransferase-encoding gene, exhibited enhanced sensitivity to exogenous sucrose and suppressed expression of the auxin-induced AUR3 gene. In cucumber, interactions between sugar and auxin were observed to induce fruit development, implying their role in fertilization process [32]. Furthermore, exogenous glucose treatment significantly upregulated the transcriptional levels of auxin biosynthetic genes in Arabidopsis [33]. Sugar levels regulated the expression of the auxin biosynthetic gene *ZmYUCCA* in developing maize grains [34]. Two auxin response factors, SlARFs in tomato, play a role in regulating sugar metabolism during tomato fruit development [35, 36]. These findings further validate the link between sugar and auxin metabolism.

The development of plant roots is extremely important throughout the life, as it enables the plant to obtain nutrients. Additionally, well-developed roots help stabilize plants in soil and protect them from external environmental influences [37]. The formation of lateral roots increases the surface area of the root system [37]. In contrast to primary roots, lateral root primordia originates from specialized procambium cells found in the middle region of the existing root. They traverse three cell layers, including the adjacent endodermis, cortex, and epidermis, until the lateral roots are generated. This entire process includes eight stages, starting from the production of lateral root primordia by procambium cells and ending with the breakthrough of the epidermis. The coordination of this process depends on the role of auxin [38, 39]. Auxin triggers the degradation of AUX/IAA repressor factors and controls the expression of target genes through auxin response transcription factors (ARFs) [39, 40]. Apart from being influenced by hormones and other internal factors, the formation of lateral roots is also regulated by external factors like nutrition and water status.

Salinity, drought, and other abiotic stresses can cause plants to produce excessive reactive oxygen species (ROS), disrupting various physiological and biochemical reactions within cells and leading to metabolic disorders or death in plants [41]. To counteract the damage caused by ROS, plants produce a series of scavengers to eliminate the antioxidant substances such as Superoxide dismutase (SOD), catalase (CAT), glutathione (GSH), and ascorbic acid (AsA), etc. Among them, AsA is one of the most abundant water-soluble antioxidants. Evidence suggests that AsA plays important roles in plant growth and development, flowering and senescence, as well as programmed cell death [42]. The phosphomannomutase (PMM), is a pivotal enzyme involved in AsA synthesis in organisms. Overexpression of the *MgPMM* gene in acerola cherry (*Malpighia glabra*) and *AtPMM* in *Arabidopsis* resulted in AsA content increase [43, 44]. Additionally, overexpression of the *DoPMM* gene in *Dendrobium officinale* enhanced the plant’s tolerance to osmotic stress in Arabidopsis.

Soybean plants (*Glycine max* ) are important oil crop. Soybean yield is affected seriously because of changes in climate, land evaporation, rise in sea level and soil salinization [45]. In order to grow in saline soil, cultivating abiotic tolerant soybean variety is important for soybean yield. In previous studies, we have characterized the soybean hexokinase gene family members and confirmed that the *GmHXK2* gene can contribute to plant growth and development under salt stress [46]. Here, we demonstrated that *GmHXK2* is significantly expressed in every tissue including leaves, stem and roots at early stage, but is specifically inhibited in petiole and petals at later stage. Root development in soybean plants with silenced *GmHXK2* was retardative along with the phenotype of slower growth. GmHXK2 was confirmed to interact with GmPMM through yeast two-hybridization and bimolecular fluorescence complementation assays. The gene *GmHXK2* plays a key role in participating in auxin synthesis and distribution, thus regulating root development, enhancing the ability to absorb water, and suppressing salt stress. These findings could be utilized in breeding programs to cultivate salt-tolerant crops in the future.

## Results

### Evolutionary analysis *HXKs* among *Glycine max*, *Arabidopsis thaliana* and *Oryza sativa*

In our previous study, MEGA-X software was used to construct a phylogenetic tree of 56 *hexokinases* from *G. max, A. thaliana, S. lycopersicum, O. sativa* and *N. tobacum* [46]. Among them, 33 members were from *G. max, A. thaliana* and *O. sativa*. The majority of *HXK*s (27 *HXKs*) out of 33 *HXKs* were grouped into Cluster III, indicating the close relationship among the three species of plant. However, other 6 members including *GmHXK1, GmHXK2, GmHXK 3, GmHXK4, OsHXK4* and *AtHXK3* were grouped into cluster II. Here, to investigate the selection pressure on *HXK* genes, we identified paralogous genes in soybean and orthologous genes among Arabidopsis, soybean and rice using bidirectional best-hit methods. The results were represented in Fig. S1A and S1B. In Table S3, we have listed their corresponding Ka/Ks values. Generally, the value of Ka and Ks respectively represents the nonsynonymous substitution rate and synonymous substitution rate. The value of Ka/Ks greater than 1, equal to 1, and less than 1 represents positive selection, neutral selection, and negative selection respectively. Our results showed that out of the 17 *GmHXKs*, 14 paralogs were found with Ka/Ks values less than 1. On the other hand, *GmHXK1*, *GmHXK2*, and *GmHXK13* displayed significant differences among *GmHXKs* family members. Analyzation on the orthologous *HXKs* genes among Arabidopsis, soybean and rice suggested that 8 *GmHXKs* showeds collinear with 3 *AtHXKs* and 3 *OsHXKs*. Furthermore, the Ka/Ks values for these genes were also found to be less than 1, indicating that they had experienced purifying selection pressure after gene duplication events.

### *GmHXK2* gene multiple sequence alignment and protein interaction analysis

**Fig. 1:**
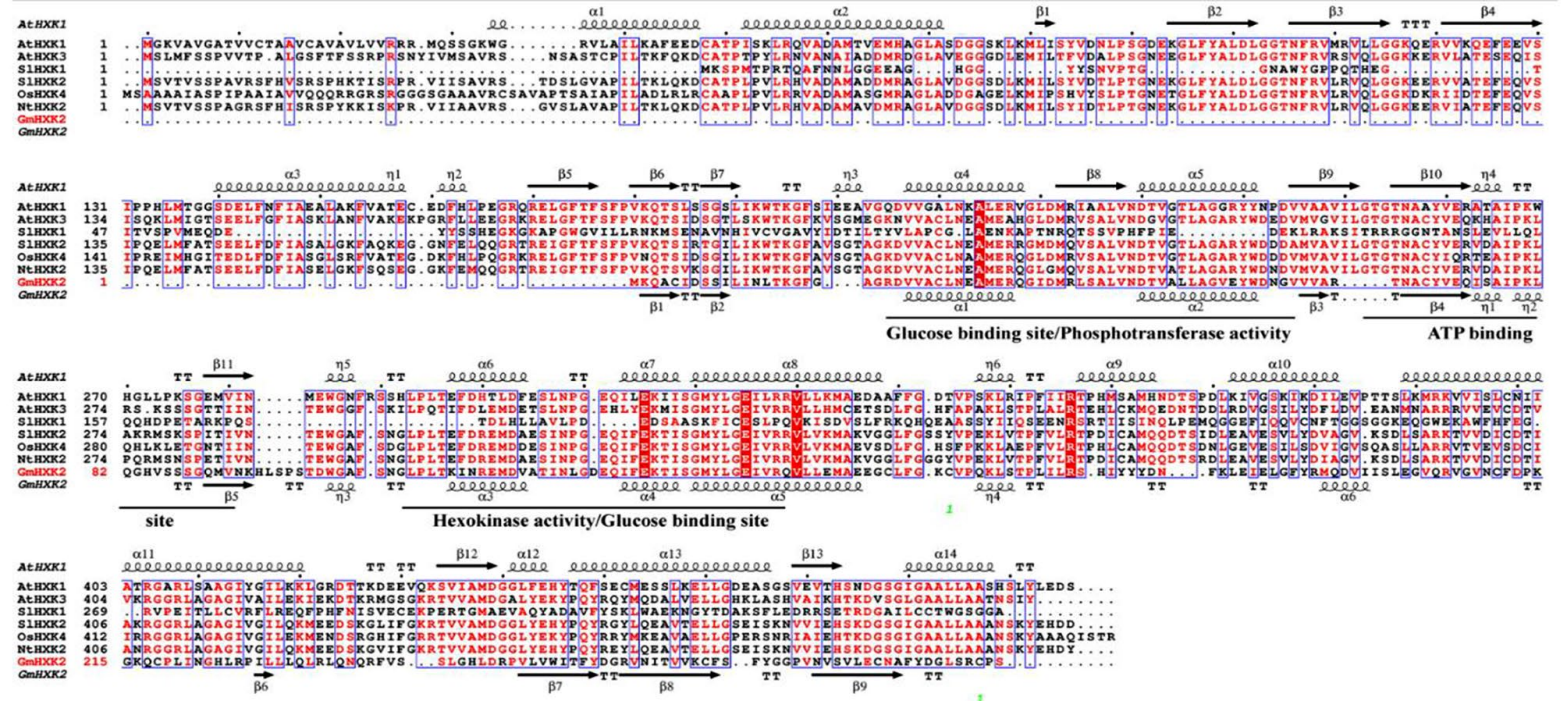
Amino acid sequence alignment among the domain of different plant species of Hexokinases. Highly conserved residues are highlighted in red shade. η represents the 3_10_-helix, the wavy line denotes the α-helix, the arrow denotes the β-fold, TT denotes the β-turn with 180° fold structure, and green numbers mark the position of the disulfide bond

Based on the previously phylogenetic tree analysis [46], multiple sequence alignment was performed among GmHXK2 protein and evolutionarily closely related proteins AtHXK3, SlHXK1, SlHXK2, OsHXK4, NtHXK2, as well as AtHXK1. As shown in Fig. 1, conserved amino residues are marked in red color, and highly conserved residues are shown in white color font along with red shade. GmHXK2 contains two disulfide bonds, three 3_10_-helices, six α-helices, nine β-folds, and twelve 180° β-turns. The conserved motifs of GmHXK2 protein include glucose binding domain, hexokinase activity domain, phosphotransferase activity domain, and ATP binding domain.

**Fig. 2:**
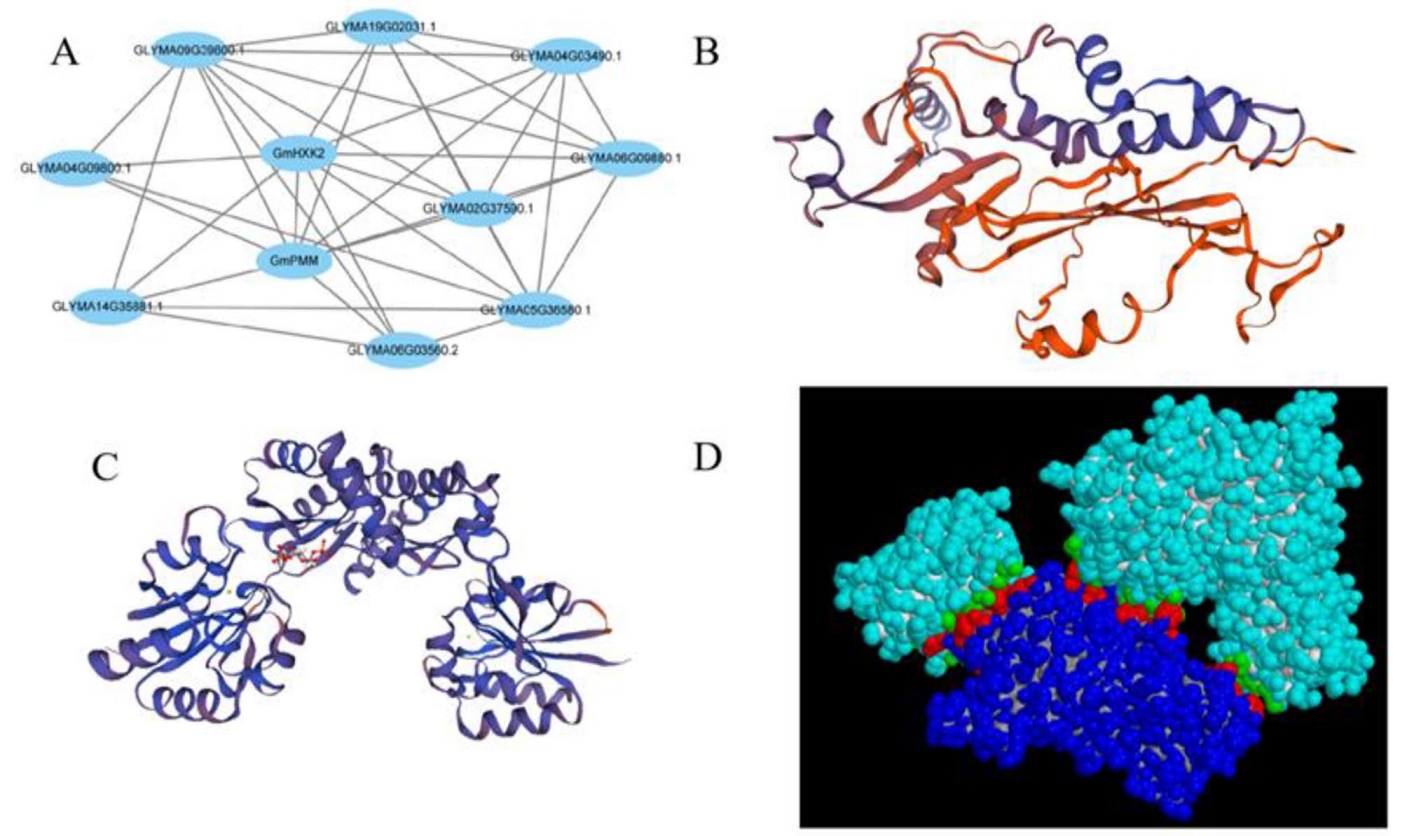
GmHXK2 protein structure and interaction analysis. (**A**) Predicted interaction network diagram of GmHXK2 protein; (**B**) GmHXK2 protein structure; (**C**) GmPMM protein structure; (**D**) Schematic diagram of the three-dimensional structure of the protein complex

The STRING database was used to evaluate target proteins interacting with GmHXK2. As shown in Fig. 2A, GmPMM has the closest affinity with GmHXK2. The tertiary structure of the GmHXK2 protein was obtained with SWISS-MODEL homology modeling(Fig. 2B). In order to analyze the interaction between GmHXK2 protein and GmPMM protein, the predicted protein-binding structure in ZDOCK was further analyzed and simulated in PDBePISA. Figure 2C shows the GmPMM protein structure simulated in SWISS-MODEL, which is modeled with a high matching degree. The protein docking complex was shown in Fig. 2D, with a free energy of -12.9 and an interaction surface area of 1932.5. The deep blue marks the GmHXK2 protein structure, the shallow blue marks the GmPMM protein, and red and green mark the binding sites of the two proteins, which is dependent on twelve hydrogen bonds and eight salt bridges.

### Analysis of the tissue-specific expression pattern of *GmHXK2*

An analysis of cis-acting elements of the promoter was conducted on the 2000 bp upstream CDS of *GmHXK2*, and the results are presented in Fig. S2. The cis-elements of *GmHXK*2 included: cell cycle regulation element, meristem expression element, and light responsive element involved in plant growth and development, stress responsive element, as well as phytohormone responsive elements, such as auxin responsive element, ABA responsive element, etc. This suggested that *GmHXK2* not only had played a pivotal role in the overall life during growth stages, but also participated in response to external environment stress.

**Fig. 3:**
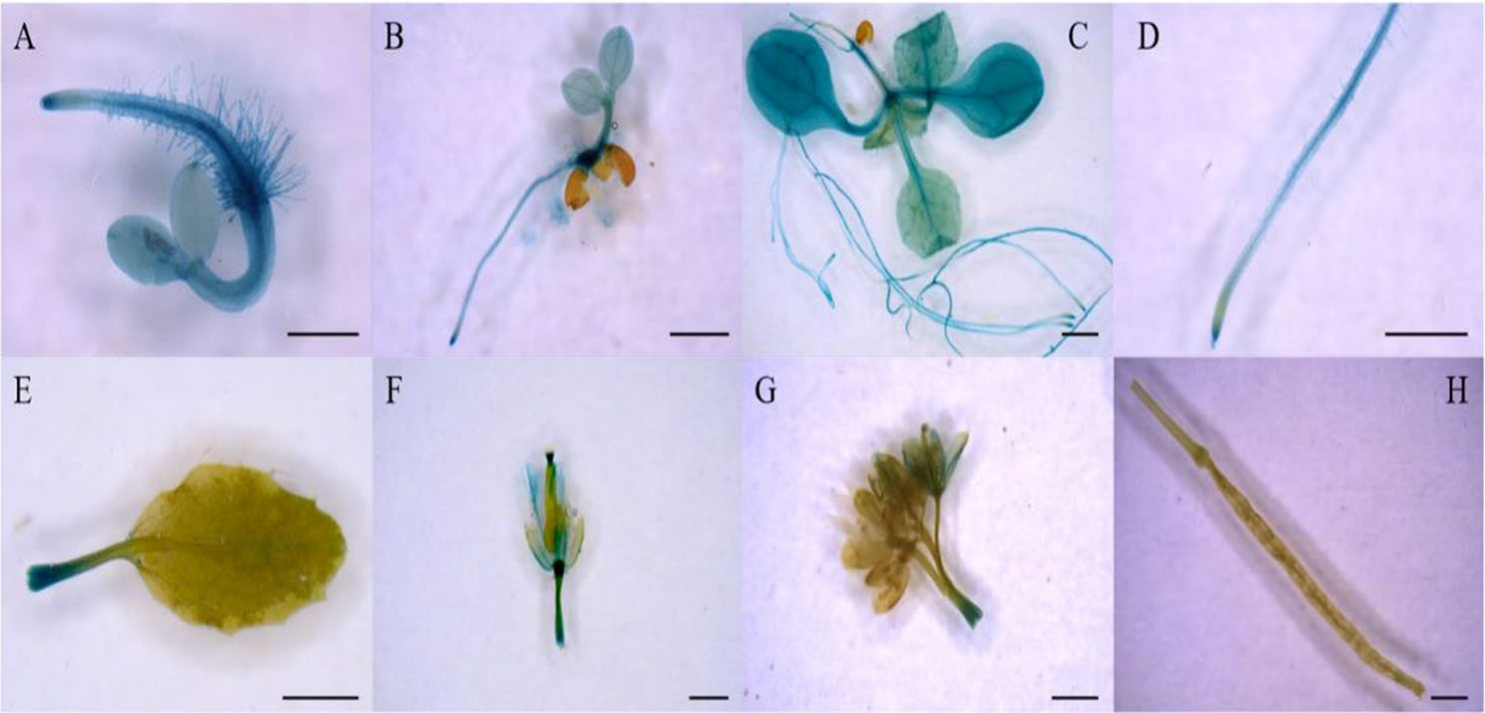
GUS staining of proGmHXK2::GUS transgenic Arabidopsis seedlings. (**A**) 3-day-old seedling; (**B**) 5-day-old seedling; (**C**) and (**D**) 14-day-old seedling leaves, primary roots; (**E**) Cotyledon leaf; (**F**) Flower; (**G**) Inflorescence; (**H**) Pod. Scale bar: A, 0.5 mm; B-H, 1 mm. GUS, β-glucuronidase

GUS staining of the T_3_ homozygous transgenic Arabidopsis expressing proGmHXK2::GUS showed that *GmHXK2* is clearly visible during the germination phase (Fig. 3A), seedling stage (Fig. 3B), and true leaf stage (Fig. 3C and D), indicating higher expression in roots、stems and leaves, and lower expression level in the petioles of the rosette leaves at early stages were detected (Fig. 3E, F and G). However, there is no expression detected in the pods and little expression level in flowers in mature period (Fig. 3H). These findings showed that *GmHXK2* mainly expressed in plant vegetative organs.

### The silencing of *GmHXK2* inhibits root development

**Fig. 4:**
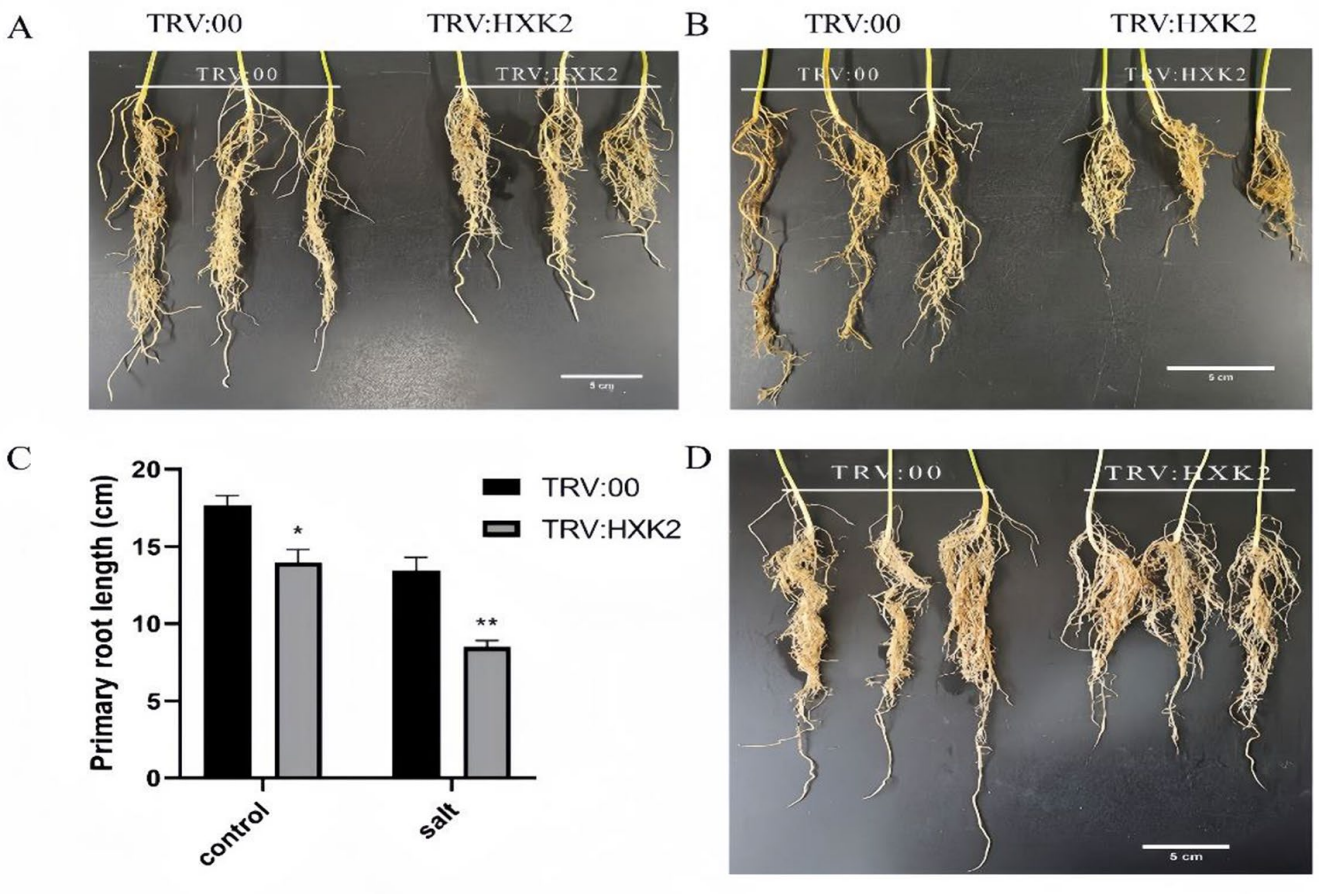
Root growth of *GmHXK2*-silenced soybean plants by VIGS technology. (**A**) The roots of *GmHXK2*-silenced soybean plants. (**B**) The roots of *GmHXK2*-silenced soybean plants with 200 mM NaCl treatment for 5 days; (**C)** The root length decreased with or without salt stress treatment. Data are presented as the mean ± SE (*: *P* < 0.05; **: *P* < 0.01; *n* = 3; t-test). (**D**) The effects of *GmHXK2* silencing on phenotype of roots development. Observation after growth for 25 days. A, B, D scale bar: 5 cm

*GmHXK2*-silencing soybean plants were obtained with VIGS (virus induced gene silencing) technology to further examine the effect of *GmHXK2* on root growth and development. The results showed that root length of *GmHXK2*-silenced soybean plants decreased compared with control plants (TRV:00) with or without 200 mM NaCl treatment for 5 days (Fig. 4A and D). In normal conditions, the roots of TRV: HXK2 displayed stunted growth than that of TRV:00 after 25 days of growth (Fig. 4D). These results indicated that the silencing of *GmHXK2* suppressed root development and reduced salt tolerance of plants.

### Effects of *GmHXK2* on synthesis and distribution of auxin

**Fig. 5:**
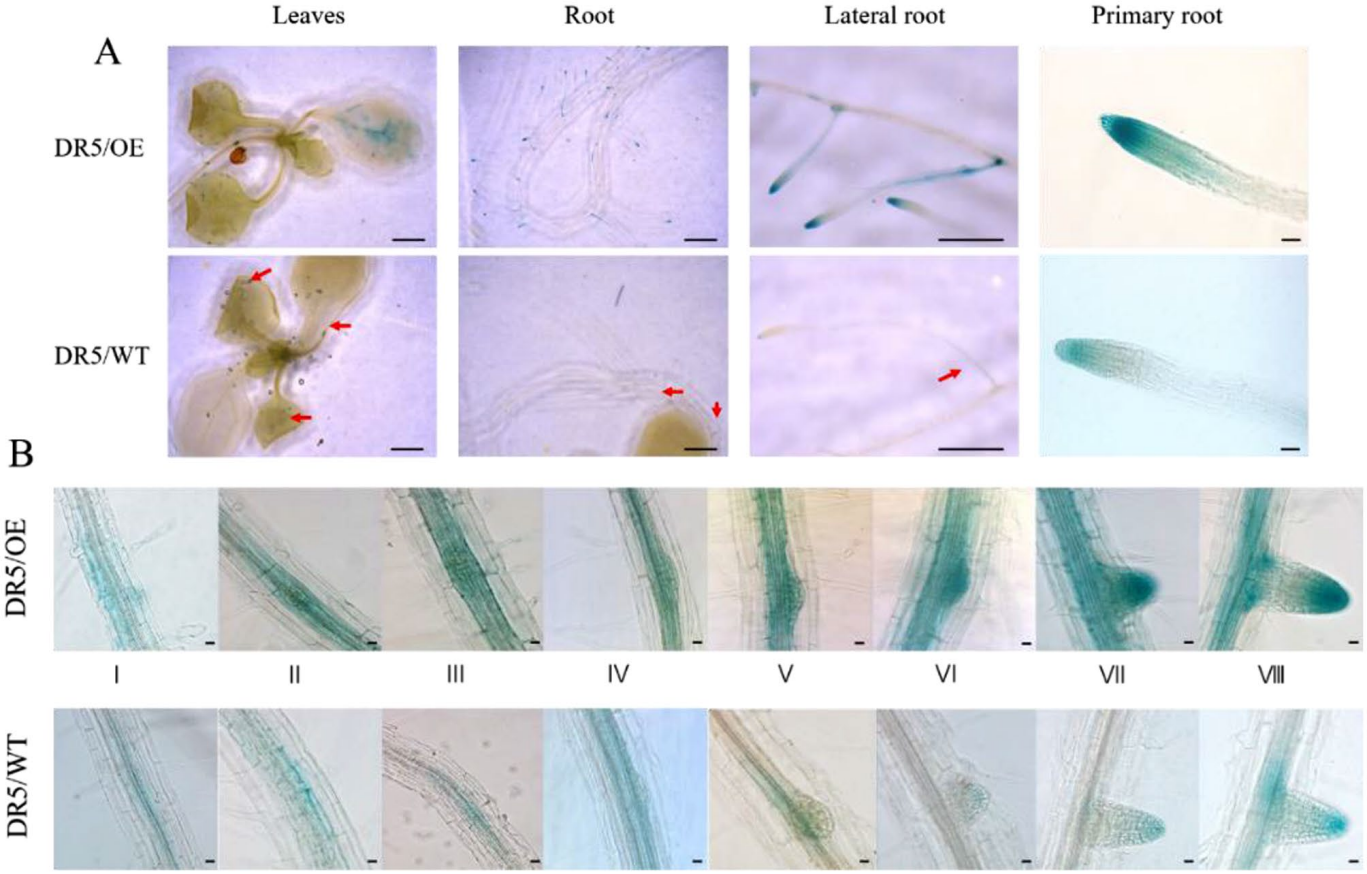
GUS Staining of DR5/OE and DR5/WT Arabidopsis plants (**A**) and their roots (**B**) under exogenous glucose treatment. Scale bar: leaves and roots 1 mm; lateral roots 500 μm; primary root 50 μm; root stage I-VIII scale: 20 μm

Auxin regulates the expansion and polarity of individual cells, as well as the initiation and patterning of organs. Transient auxin concentration gradients underlie the developmental processes such as meristem initiation, organ primordia formation, embryo morphogenesis, lateral root formation, as well as the regulation of phyllotaxy and vascular tissue differentiation, photo- and gravitropic responses [47]. Here, we assessed the potential roles of *GmHXK2* in participating in synthesis and distribution of IAA through evaluating the activity of the auxin-responsive reporters DR5::GUS in DR5/OE Arabidopsis lines. As shown in Fig. 5A, in OE plants, GUS expression was mainly concentrated in leaf veins, root tips and lateral roots, indicating that the activity of auxin response reporter DR5 in overexpressing *GmHXK2* Arabidopsis in leaves and roots (including primary and lateral roots) was up-regulated compared with that of WT. In particular, under treatment with 100 mM exogenous glucose, GUS expressions in OE plants appeared to be concentrated at the tip of the newly formed lateral roots, implying that there were more auxin content at the all stages I-VIII during lateral root development in comparison with that of the DR5/WT plants (Fig. 5-B). These results suggest that in the presence of external glucose, *GmHXK2* can induce and promote auxin synthesis and regulate its distribution in lateral roots.

**Fig. 6:**
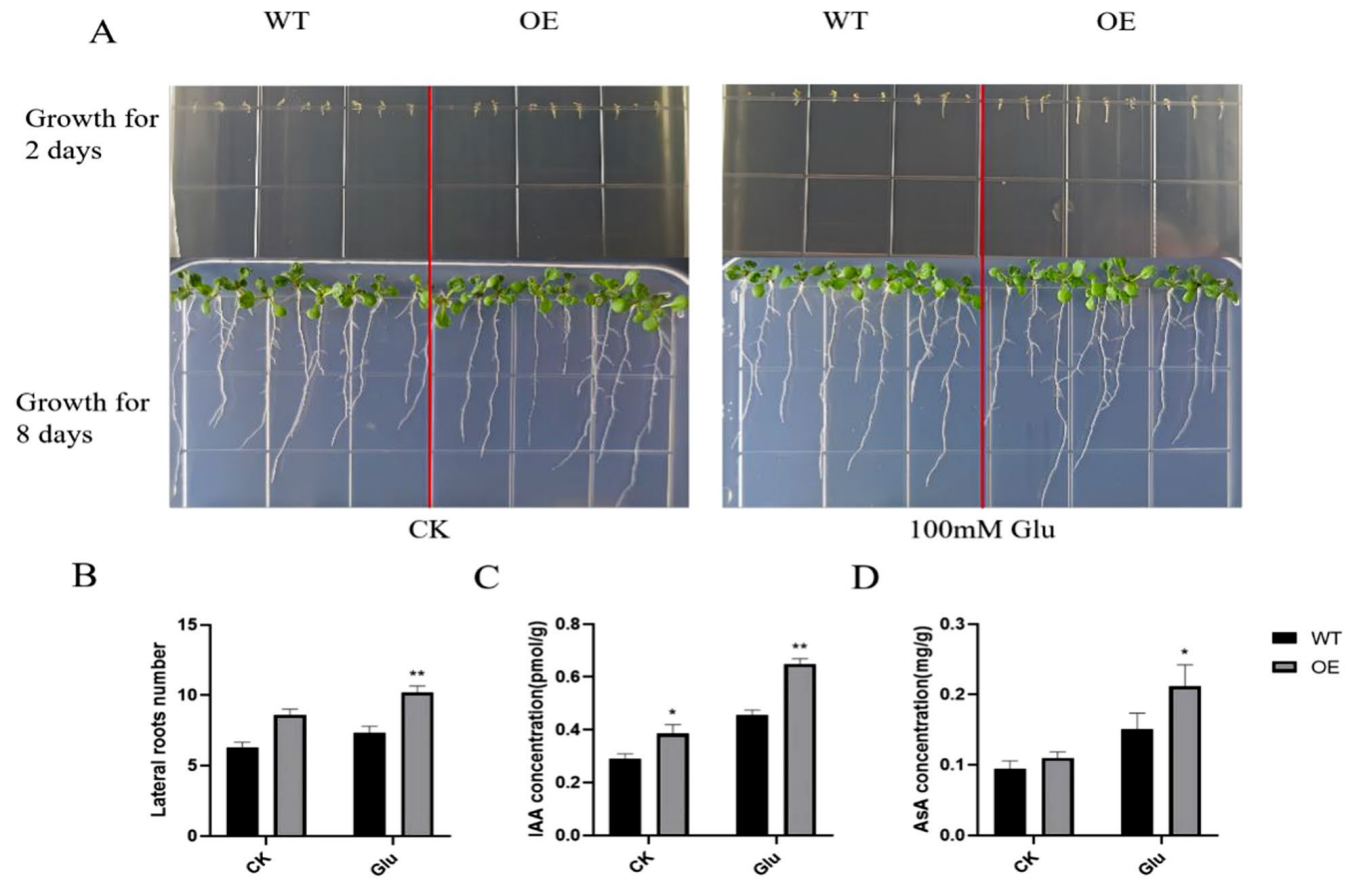
Effects of exogenous glucose on OE and WT Arabidopsis lateral root growth and IAA and AsA contents. (**A)** Lateral roots in OE and WT Arabidopsis plants under normal conditions or under treatment with 100 mM Glu; (**B)** Lateral root number in 8-day-old Arabidopsis plants; (**C)** Measurement of endogenous IAA content; (**D)** Measurement of AsA content. Statistics are shown as the mean ± SE. (*: *P* < 0.05; **: *P* < 0.01; t-test; *n* = 18)

To analyze further the relationship between *GmHXK2* and auxin biosynthesis and distribution of roots, we measured the content of auxin and the growth of roots in Arabidopsis plants. The OE and WT *A. thaliana* plants grown on the medium were observed and their growth statistics were made (Fig. 6A). With glucose treatment for 2 days, the OE plants grew faster and the root length was longer than that of WT, while there was no significant difference in the control group. After 8 days of growth, the root length of OE plants was longer than that of WT, and the number of lateral roots produced was also more than that of WT, and the number of lateral roots was more under the condition of exogenous glucose. The number of lateral roots in the OE plants increased by 37.2% and 39.4% than that in WT plants without or with exogenous glucose for 8 days, respectively (Fig. 6B). Correspondingly, the endogenous IAA content (Fig. 6C) and AsA content (Fig. 6D) in the OE plants were detected, which increased by 33.5% and 16.2% without exogenous glucose treatment, compared with that in WT plants respectively. After exogenous glucose treatment, IAA and AsA contents of OE plants were significantly increased compared with WT plants, by 42.6% and 40.5%, respectively. These results indicated that the *GmHXK2* gene participates in root development, promotes lateral root production, and enhances AsA synthesis in plants under exogenous glucose presence.

### *GmHXK2* regulates the expression of genes related to auxin synthesis and transport

**Fig. 7:**
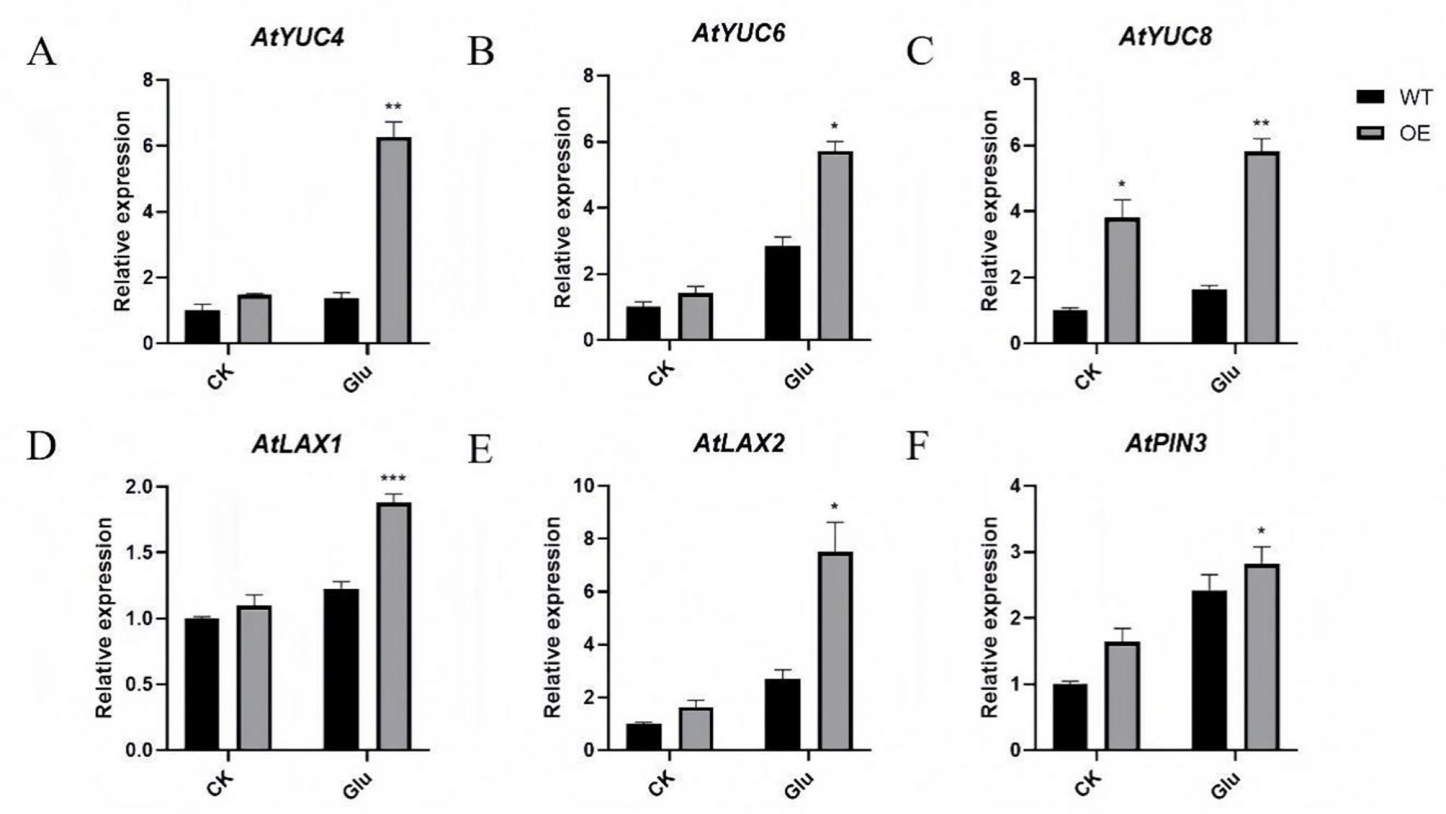
Expression of auxin-related genes in OE and WT Arabidopsis plants at conditions with exogenous glucose. (**A**)-(**C**) *AtYUC4,6,8* respectively; (**D**) *AtLAX1*; (**E**) *AtLAX2*; (**F**) *AtPIN3*. Data are presented as the mean ± SE. (*: *P* < 0.05; **: *P* < 0.01; ***: *P* < 0.001; *n* = 3; t-test)

The expression of genes related to auxin synthesis and transport is shown in Fig. 7. When treated with exogenous glucose, the expression levels of genes involved in auxin synthesis (*AtYUC4,6,8*), auxin influx (*AtLAX1,2*), and auxin efflux (*AtPIN3*), were significantly up-regulated in OE plants compared with WT plants. This finding was consistent with the observation from staining of DR5/OE seedlings, suggesting that glucose participated in regulating genes related to auxin signal transduction pathway through *GmHXK2*. As a result, the internal auxin content in plant cells increases, leading to the induction of lateral root formation and promotion of root elongation.

### Overexpression of *GmHXK2* can enhance salt tolerance in Arabidopsis plants

**Fig. 8:**
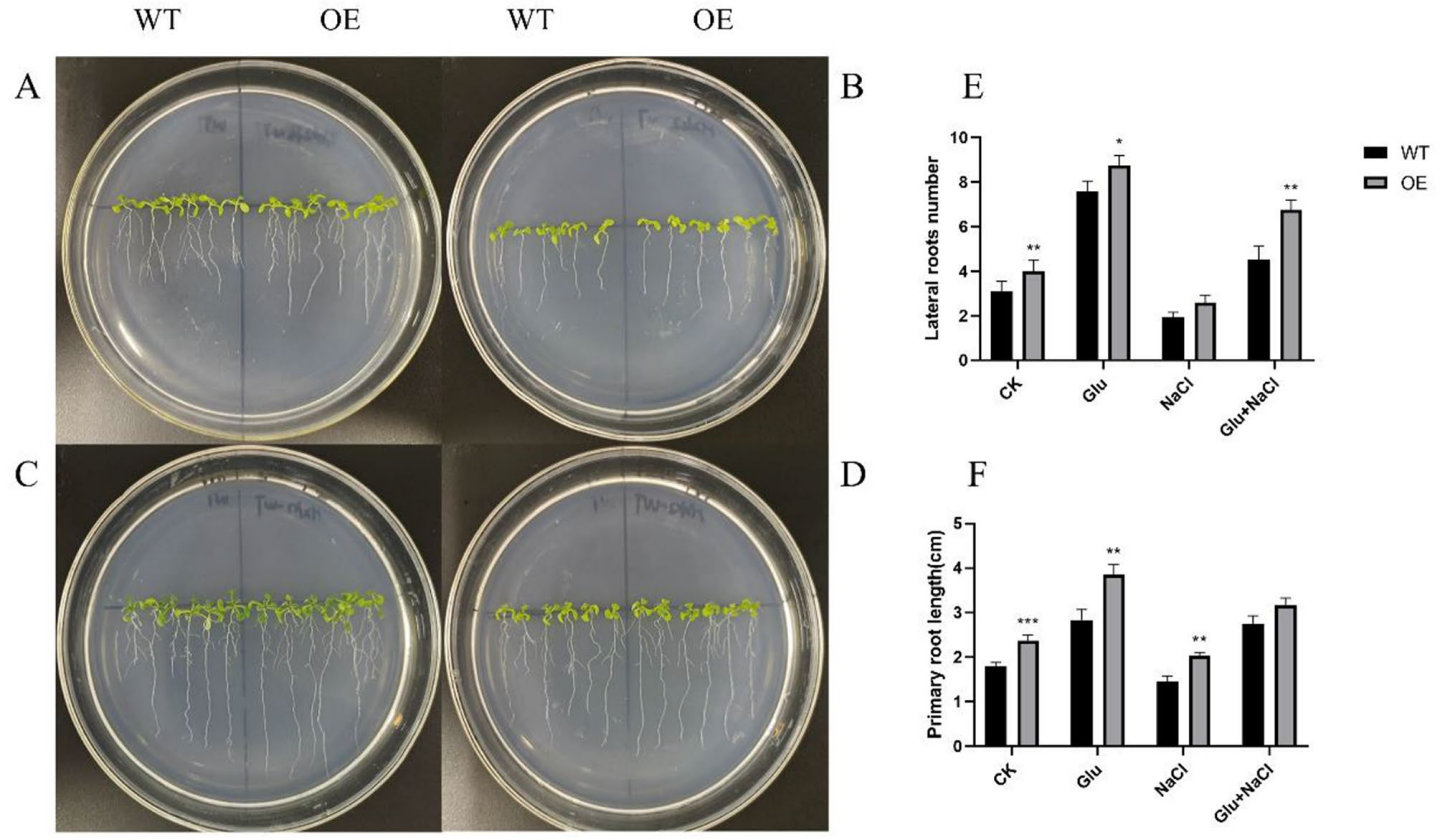
Phenotypic and root data statistics under salt and sugar treatments. (**A**) control; (**B**) 100 mM NaCl; (**C**) 100 mM Glu; (**D**) 100 mM Glu + NaCl; (**E**) lateral root number statistics; (**F**) primary root length statistics. Data are presented as mean ± SE. (*: *P* < 0.05; **: *P* < 0.01; ***: *P* < 0.001; *n* = 12; t-test)

The phenotypes of OE and WT under the conditions of glucose and NaCl treatment are shown in Fig. 8A-D, in which exogenous glucose has the best growth status, the number of lateral roots and root length are optimal, and the growth is inhibited under salt treatment. Meanwhile, the application of exogenous glucose can significantly alleviate the inhibitory effect of salt stress on growth. The number of lateral roots (Fig. 8E) and taproot length (Fig. 8F) of OE plants were superior to those of WT plants under any treatment.

**Fig. 9:**
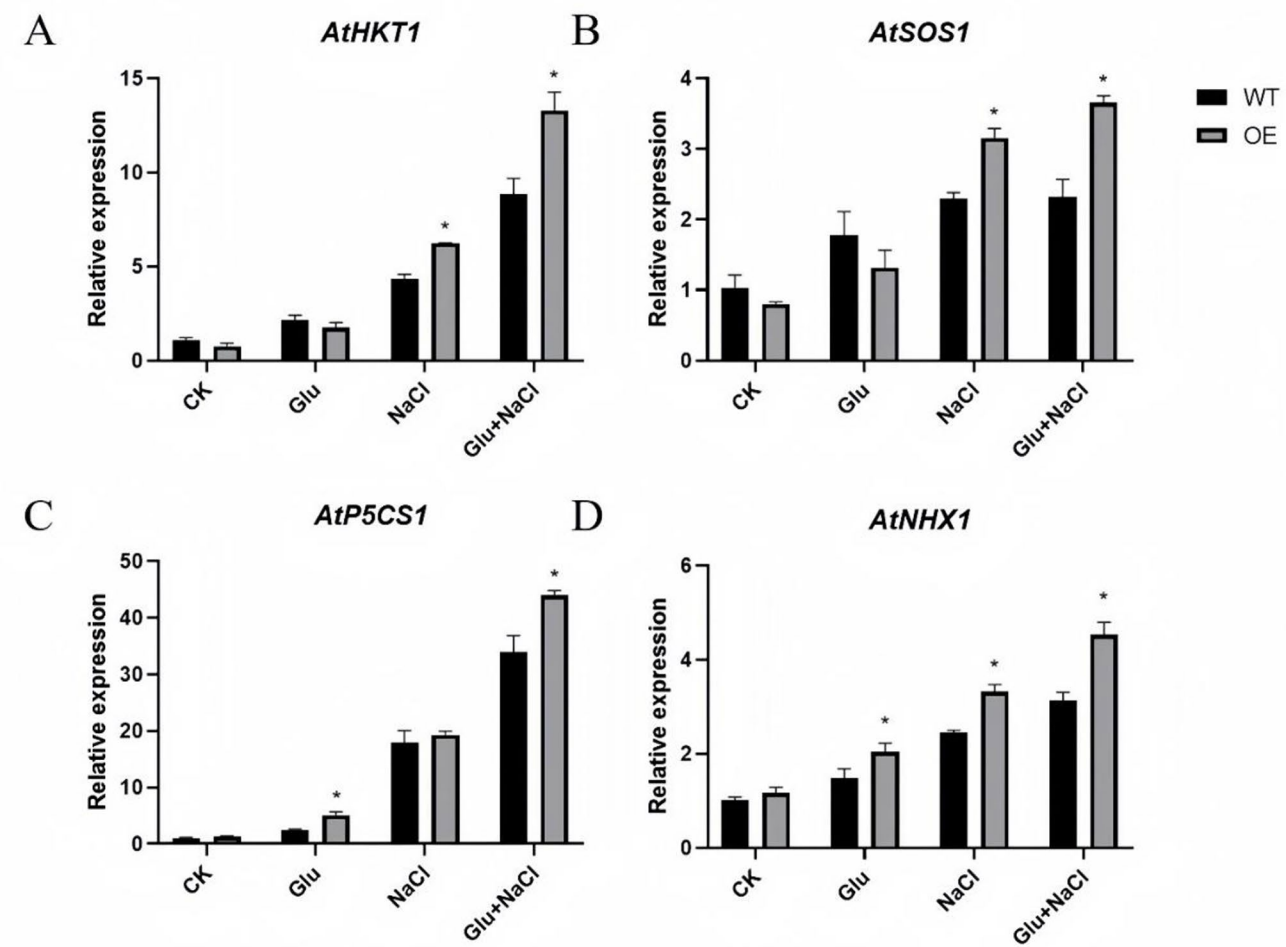
Expression levels of salt-tolerant genes in OE and WT Arabidopsis plants under salt stress. (**A**) *AtHKT1*; (**B**) *AtSOS1*; (**C**) *AtP5CS1*; (**D**) *AtNHX1*. Data are presented as the mean ± SE. (*: *P* < 0.05; *n* = 3; t-test)

Our previous report showed that *GmHXK2* can enhance salt tolerance in Arabidopsis, especially at conditions with exogenous glucose [46]. Here, we measured the expression of stress-related genes. The results showed that (Fig. 9) the expression of *AtHKT1* and *AtSOS1* in OE plants increased significantly compared with WT plants under glucose treatment. The expression of *AtP5CS1* and *AtNHX1* increased in OE obviously than that in WT plants without or with glucose. These findings suggest that *GmHXK2* plays a pivotal role in resistance to salt stress by promoting ion transport and regulating osmotic potential, which were mainly dependent on glucose signal.

### GmPMM interacts with GmHXK2 and is involved in plant stress resistance

**Fig. 10:**
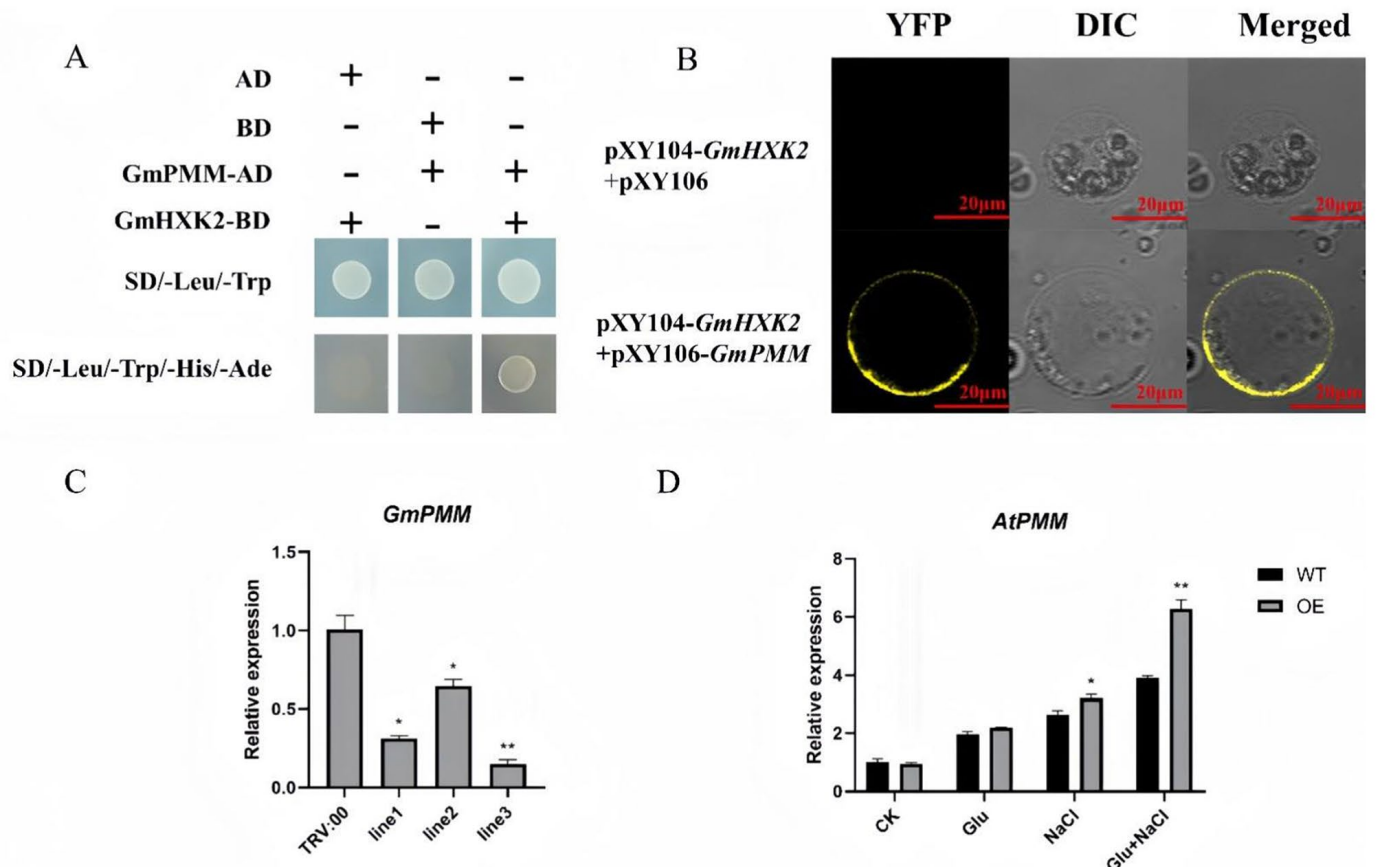
Interaction between GmHXK2 and GmPMM. (**A**) Yeast two-hybridization assay; (**B**) Bimolecular fluorescence complementation assay (Scale bar, 20 μm); (**C**) Expression levels of *GmPMM* gene in silenced soybean plants; (**D**) Expression levels of *AtPMM* gene in WT and OE Arabidopsis under different treatments. Data are presented as the mean ± SE. (*: *P* < 0.05; **: *P* < 0.01; *n* = 3; t-test)

The GmPMM and GmHXK2 showed no self-activation activity in yeast cells (Fig. 10A). Colonies successfully appeared on SD/-Leu/-Trp. However, when pGADT7 + GmHXK2-pGBKT7 and pGBKT7 + GmPMM- pGADT7 were introduced respectively, colonies did not appear on SD/-Ade/-His/-Leu/-Trp, indicating no self-activation. When the two recombinant vectors, GmPMM-pGADT7 and GmHXK2- pGBKT7, were co-transformed, the colonies appeared on SD/-Leu/-Trp and SD/-Ade/-His/-Leu/-Trp media, demonstrating the interaction between GmPMM and GmHXK2 in vitro. To verify the interaction in vivo, we performed BiFC assay in Nicotiana, as depicted in Fig. 10B. The negative control group pXY104-GmHXK2 + pXY106 did not elicit any fluorescence, while the experimental group (pXY104-GmHXK2 + pXY106-GmPMM) showed yellow fluorescence signal in the plasma membrane.

The expression of *PMM* in soybean-silenced plants (Fig. 10C) and OE Arabidopsis (Fig. 10D) under salt stress were analyzed further. The expression of *GmPMM* was detected to be down-regulated in soybean-silenced *GmHXK2* plants compared with the wild type. Interestingly, no significant difference in the expression of the *AtPMM* gene was detected between the OE and WT at normal conditions. However, under NaCl treatment, *AtPMM* gene displayed up-regulated expression in both WT and OE lines. Notably, the *AtPMM* in OE plants exhibited significantly upregulation compared with WT, especially under glucose treatment, indicating that *GmHXK2* expression in *Arabidopsis* can promote the expression of the *AtPMM* gene. This enhanced AsA synthesis, which maybe improved the plant antioxidant capacity, and ultimately enhanced salt tolerance.

## Discussion

Studies on the function of hexokinase have shown that hexokinase is widely involved in plant development and response to environmental stress [21, 23, 48]. As a glucose sensor, hexokinase is associated with nutrient, light and various hormone signaling networks [31]. It relies on the hexokinase signaling pathway to crosstalk with multiple hormone signaling pathways and mediates the molecular mechanisms underlying various developmental and physiological processes [49]. *AtHXK1* has dual functions in glucose signaling and metabolism, and closely interacts with auxin and cytokinin signaling pathways [31, 50]. The promoter of *GmHXK2* contains ABA, auxin, and stress response elements, implying that it may respond to a variety of signals (Fig. S2). Our results suggest that GmHXK2 acts as a glucose sensor, connecting sugar signaling and auxin signaling, thereby promoting root development.

Abiotic stress disrupts metabolic processes in plants, leading to excessive production of ROS in plant cells, thus damaging cell structure and biological function. AsA is an effective substance produced by plants to combat oxidative stress. Here, we confirmed that GmPMM can interact with GmHXK2 through yeast two-hybridization and BiFC. Overexpression of *PMM* gene in cherry [43], *Dendrobium officinale* [51], *A. thaliana* [44], and rice can all increase AsA content in plants. Here, we demonstrated that GmHXK2 can influence the expression of PMM in soybean and Arabidopsis, and promote the increase of AsA content in plants. AsA is not only a well-known antioxidant molecule in plants that has a protective effect on ROS, but also a key component in plants involved in controlling cell division and cell expansion [52]. Furthermore, molecular genetic studies have revealed the regulatory role of AsA in cell wall synthesis. Compared with *Col-0*, the cell proliferation and root growth of the AsA pathway mutant *vtc1-1* were significantly decreased [52]. This suggests that the glucose signaling pathway also interacts with the AsA signaling pathway to work together against ROS and promote the development of OE plant roots.

Apple MdHXK1 can phosphorylate and interact with Ser-275 residue of salt-stressed protein MdNHX1, thereby increasing the expression of *MdNHX1* gene and enhancing salt tolerance [23]. In Arabidopsis, the calcium-binding protein CML10, which mediates cellular physiological processes, interacts with PMM by binding Ca^2+^ to its EF-hand motif binding site [53], increasing PMM activity and enhancing AsA content. Our results showed that GmHXK2 could interact with GmPMM protein to promote the production of AsA. It is conferred that Ca^2+^ signal and sugar signal crosstalk each other, and promote the production of AsA.

The development of lateral roots in plants is a complex process in which auxin plays a crucial role. The occurrence of lateral root primordia is related to the concentration gradient of root auxin. This process depends on auxin efflux transporters PIN and auxin influx transporters AUX1/LAX for auxin transport. Auxin biosynthesis is mainly carried out through the tryptophan dependent pathway (TRP), and the *YUCCA* gene family is the key node of this pathway [54]. PIN is a membrane-bound transporter that can polally transport auxin and transmit signals, thereby regulating intracellular auxin concentration. Studies have reported that *PIN3* gene plays an important role in lateral root development and is regulated by auxin signaling [29, 55]. TAUX/LAXs are the major carriers involved in auxin influx and can also regulate root development and lateral root formation [25]. Yang et al. demonstrated that B1L interacts with the exocyst to regulate PIN-mediated polar auxin transport and lateral root initiation in Arabidopsis [56]. The study by Gupta et al. suggests that under natural environmental conditions, modulation of endogenous sugar levels can manipulate root architecture for optimized development by altering its nutrient/water uptake as well as its anchorage capacity [57]. Here, with the application of glucose, the number of lateral roots and IAA content of OE plants increased, and the expressions of *YUC4-8, LAX1/LAX2* and *PIN3* were upregated, indicating that *GmHXK2* was involved in the biosynthesis and distribution of IAA in the root system, thus promoting the development of taproot and lateral roots.

Plants regulate the expression of salt stress response genes through a series of signal transduction to maintain osmotic balance under salt stress. HKT1, SOS1, and NHX1 are ion transporters located on the cell membrane [58]. Under high salt conditions, they transport Na^+^ or K^+^ to maintain the ion balance within the cell, reducing the damage caused by ion toxicity to the plant. HKT1 is an important carrier of Na^+^ in cells. Under salt stress, the affinity of HKT1 to Na^+^ is much higher than that of K^+^, thus inhibiting the transport of Na^+^ to the above-ground part and protecting plants from ionic toxicity [59, 60]. *SOS1* is a key gene in the SOS regulatory pathway and plays an important role in the regulation and transport of Na^+^ [61, 62]. NHX1, as a sodium-proton exchanger protein, participates in ion transport and salt tolerance of plant cells by consuming ATP to transport Na^+^, thus maintaining osmotic balance [63]. *P5CS1* is a key gene in proline biosynthesis pathway. The increase of proline content can balance the osmotic potential of cells, weaken the effect of osmotic stress on plants, and play a positive regulating role in plant salt tolerance [55, 63]. In OE plants, the expressions of *HKT1*, *SOS1*, *NHX1* and *P5CS1* were increased, and exogenous glucose further promoted their expression under salt stress (Fig. 9). These results suggest that *GmHXK2* gene not only regulates root development and promotes growth, but also acts as a glucose sensor to promote salt tolerance by maintaining intracellular ion balance and reducing damage under salt stress.

**Fig. 11:**
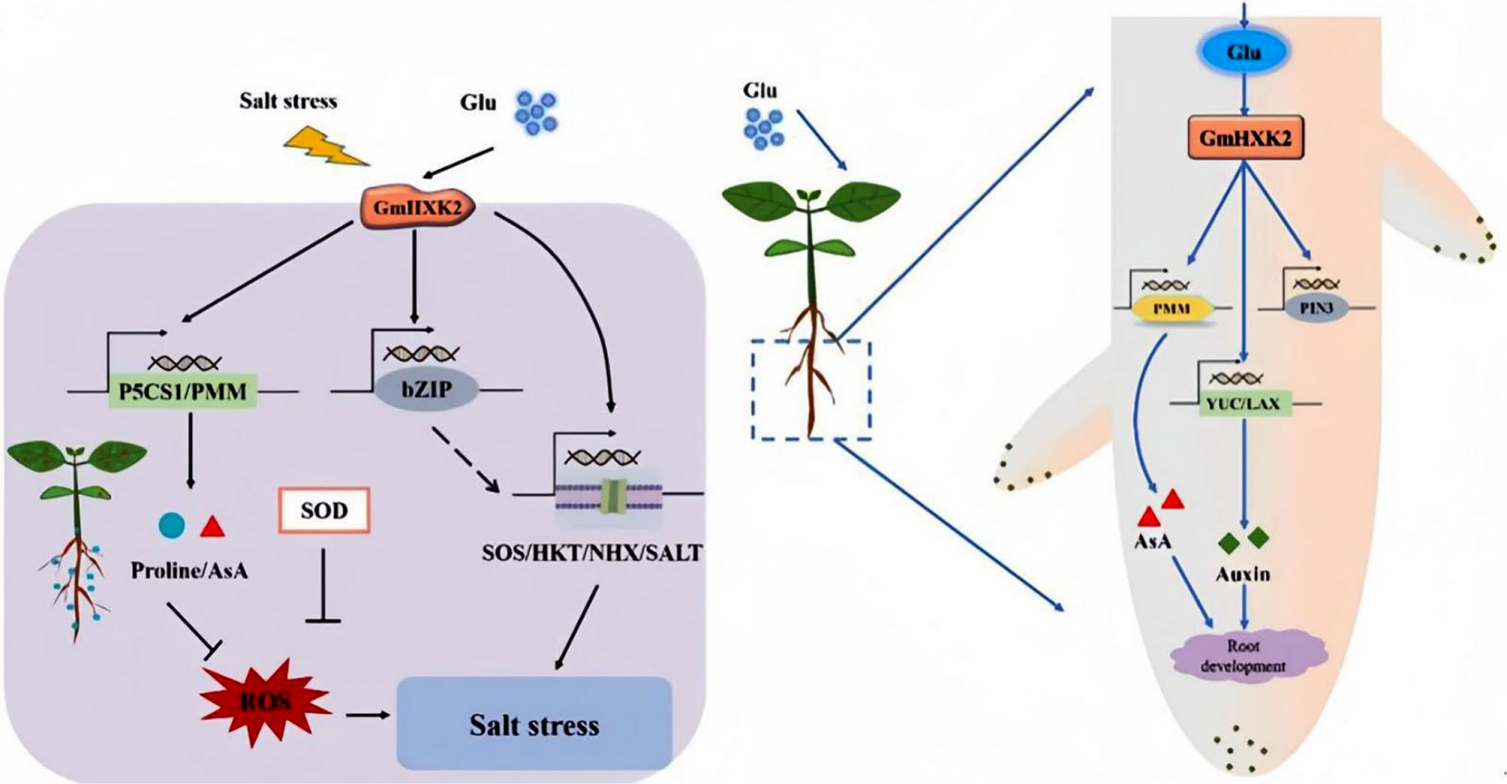
Molecular mechanism scheme of *GmHXK2* to improve plant salt tolerance and root development. Under salt stress, GmHXK2 regulates the expression of downstream bZIP transcription factors, which promote the expression of *SOS, HKT, NHX* and *SALT, P5CS* and *PMM*, thereby improving the antioxidant capacity and salt tolerance of plants (**Left**). Under the application of exogenous glucose, GmHXK2 can further crosstalk with sugar signaling to promote IAA synthesis and IAA redistribution, and thus regulate the expression of genes related to root development. In addition, GmHXK2 interacts with PMM to promote AsA synthesis and improve tolerance to salt stress (**Right**)

A schematic diagram was established as shown in Fig. 11. *GmHXK2* plays an active role in regulating auxin synthesis and distribution, thus promoting root development and plant growth. Under salt stress, GmHXK2 not only interacts with GmPMM to promote AsA synthesis, eliminate ROS, and promote root growth, but also regulates the expression of salt stress response genes, reduces the toxicity of Na^+^, and maintains osmotic balance.

The molecular mechanism of plant stress resistance is one of the eternal themes in plant science. Recently, researchers have made exciting progress from different perspectives. In the past, many studies focused on the role of transcription factors (TFs) in abiotic stress, because they can regulate the expression of a large number of downstream target genes. For example, *GmNAC3* and *GmCAMTA12* genes have a potential role in regulating the response of soybeans to drought stress [64, 65]. Similarly, *CsWRKY29* and *CsWRKY37* in tea plants confer cold tolerance in transgenic Arabidopsis [66]. In recent researches, long noncoding-RNAs (lncRNAs) and circular RNAs (circRNAs) were identified in soybeans, highlighting their potential contribution to abiotic stress responses [67]. Additionally, either metabolites, including flavonoids [68] and anthocyanins [69–71], or macromolecular polymers, including lignin [72] and polyethylene glycol [73], all play roles in plant response to various abiotic stresses. Regulation of hormones also helps promote abiotic tolerance. *COP1* [74] and *SmCOP1LIKE* [75] can affect ethylene signaling in tomato and respond to abiotic stress. Inhibition of *SlGRAS15* leads to a series of developmental processes by regulating gibberellin signaling [76]. Here, we found that the metabolite AsA is involved in the response of soybeans to salt tolerance. And IAA signaling promoted plant salt tolerance through root development, which in turn increased water and nutrient intake of plants. Characterizing hub genes involved in AsA metabolic processes and IAA signaling pathways in plants, and regulatory networks has broadened our understanding of the mechanisms of plant stress resistance.

## Conclusion

HXKs are key enzymes in glycolysis and sugar signaling pathways in plants. The expression of *GmHXK2* gene is organ-specific and developmental stage (vegetative growth and reproductive growth) specific in plants. GmHXK2 can affect the expression of *PMM* gene in soybean and *A. thaliana* and interact with PMM protein. *GmHXK2* can not only regulate AsA synthesis and auxin synthesis and distribution, but also promote root elongation, induce lateral root formation, and potentially enhance soil water absorption by plants. Our study reveals the crosstalk between sugar signaling and hormone signaling in plants, with HXKs acting as a glucose sensor, and sheds light on the molecular mechanism by which the *GmHXK2* gene promotes salt tolerance in plants. We believe that the identification and functional analysis of hub genes in the regulatory network, constructed by the crosstalks among different signaling pathways, will deepen our understanding of the molecular mechanism of plant stress resistance, and provide us with new ideas and strategies to improve tolerance to abiotic environment of crops.

## Methods

### Bioinformatics analysis of GmHXK2

Genomic data and protein annotation of soybean, Arabidopsis, and rice from the NCBI database was downloaded (https://www.ncbi.nlm.nih.gov/). Tbtools (https://github.com/CJ-Chen/TBtools/releases) were used for BLAST analysis to generate gene collinearity maps, and Ka/Ks was calculated. The protein interaction network of GmHXK2 was obtained using STRING (https://cn.string-db.org/). SWISS-MODEL and the PDB database (https://www.rcsb.org/) was used to perform prediction on tertiary structure of protein, and ZDOCK SERVER (https://zdock.umassmed.edu/) was used for protein docking. MEGA X (https://www.megasoftware.net/dload_win_gui) is used for alignment between homologous genes and secondary structure visualization are obtained using Espript3.0 (https://espript.ibcp.fr/ESPript/ESPript/). Promoter analysis of *GmHXK2* is performed using PlantCARE (http://bioinformatics.psb.ugent.be/webtools/plantcare/htmL/).

### Tissue-specific expression of *GmHXK2*

Soybean (Williams) seeds were placed on petri dish for germination and then planted in soil in a greenhouse with photoperiod of 16 h/8 hrs at temperature 28^0^C/24^0^C in day/night until the first leave fully expanded. Genomic DNA was extracted from soybean plants, and the promoter fragment of *GmHXK2* was amplified by PCR ( the primers was listed in Table S1). The amplified fragment was inserted into the pCAMBIA3301 to construct the proGmHXK2::GUS vector, which was then transformed into wild-type Arabidopsis by dipping flowers methods. The harvested seeds were screened with Basta herbicide. Transgenic plants were identified until the T_3_ generation. The seeds of T_3_ plants were sown on 1/2MS medium and the seedlings at different growth stages were stained with GUS staining reagent (Solarbio, China) and observed by ZEISS Smartzoom 5 microscope.

### Obtaining transgenic Arabidopsis with DR5::GUS

After the DR5 promoter fragment was synthesized and inserted into the pCAMBIA3301 vector, the vectors were transformed into Arabidopsis WT and OE (overexpressing *GmHXK2* Arabidopsis) plants, respectively, using the flower dipping method. The obtained transgenic plants were named after DR5/WT and DR5/OE respectively. DR5/OE and DR5/WT seeds were further planted for screening until the T_3_ generation. Development of root and auxin distribution of *A. thaliana* expressing *GmHXK2* were assayed under NaCl treatment.

The homozygous transgenic T_3_ plants were sown on 1/2 MS and 1/2 MS supplemented with 100 mM Glu plates, respectively. After growing for 5 days in an illuminated incubator, they were stained for analysis of auxin distribution using microscopes. The plants were observed, and photographs were taken on the 2nd and 8th days. The contents of auxin and AsA were determined by ELISA reagent kits (Jiancheng, Nanjing). Additionally, some homozygous T_3_ plants were transferred onto 1/2 MS medium containing 100 mM Glu, 100 mM NaCl, and 100 mM Glu + 100 mM NaCl for 9 days. Their mRNA samples were extracted for qRT-PCR experiments.

### Induction of *GmHXK2* silencing by VIGS

The *GmHXK2* fragment was amplified with specific primers and then inserted into the pTRV2 vector. Soybean seeds were infected to obtain *GmHXK2* silenced soybean plants. The primer sequences are listed in Table S1. The experimental procedures were the same as those described in our previous report [46]. The root development and growth of silenced soybean plants (TRV: HXK2) were observed and measured.

### Yeast two-hybridization assay

The open reading frame of *GmHXK2* and *GmPMM* gene were amplified by PCR with the primer pairs (Table S1). The PCR product of *GmHXK2* was digested with restriction enzyme *Nde* I and *BamH* I, and then ligated into restriction enzyme *Nde* I and *BamH* I sites of the pGBKT7 bait vector. Similarly, the PCR product of *GmPMM* was digested with restriction enzyme *Nde* I and *BamH* I, and then ligated into restriction enzyme *Nde* I and *BamH* I sites of the pGADT7 prey vector. Subsequently, the pGBKT7 + GmPMM-pGADT7 vector and pGADT7 + GmHXK2- pGBKT7 vector were co-transformed into Y2HGold yeast competent cells respectively following the manufacturer’s instructions (Zoman, Beijing). Then the transformed mixture was spread on SD/-Leu/-Trp and SD/-Leu/-Trp/-His/-Ade media to observe colony growth for estimation of the self-activation of the GmPMM or GmHXK2. The GmHXK2-pGBKT7 + GmPMM-pGADT7 recombinant vector was further co-transformed into yeast competent cells to validate the interaction between GmPMM and GmHXK2.

### Interaction of GmHXK2 and GmPMM in vivo by bimolecular fluorescence complementation (BiFC) in *Nicotiana benthamiana*

To analyze the interaction of GmHXK2 and GmPMM in vivo, the PCR products of *GmHXK2* and *GmPMM* were fused into frame of N-terminal fragment of YFP (pXY106-nYFP) and the C-terminal fragment of the YFP (pXY104-cYFP) respectively (the primer sequences used for BiFC are listed in Table S2). The pXY104-GmHXK2 and the pXY106-GmPMM (with terminator) recombinant vectors were co-transformed into *Agrobacterium* (GV3101) by means of Agrobacterium-mediated infiltration [77]. These plasmids were co-expressed in *N. benthamiana.* The mixture containing *Agrobacterium* cells were injected tobacco leaves [78]. Then the injected tobacco leaves were cut into strips with 1 mm-wide and placed in an enzyme solution (0.2 g cellulase, 0.1 g pectinase, 0.222 g CaCl_2_, 1.0932 g mannitol, 0.1066 g MES, dissolved in 10 mL water) for digestion for 3 h. The yellow fluorescence was visualized under a confocal laser scanning microscope (ZEISS).

qRT-PCR experiment and data statistical analysis.

The primers for the qRT-PCR experiments were listed in Table S4. All samples were taken for three independent replicates. GraphPad Prism 8 was used for data analysis. The significance of the data was analyzed using Student’s t-test [56]. Data are presented as mean ± SE. Single, double, and three asterisks denote significant differences in comparison with the values of WT at *p* < 0.05, *p* < 0.01, and *p* < 0.001, respectively.

### Electronic supplementary material

Below is the link to the electronic supplementary material.

Supplementary Material 1

Supplementary Material 2

Supplementary Material 3

Supplementary Material 4

Supplementary Material 5

Supplementary Material 6

## Data Availability

The original contributions presented in the study are included in the article/Supplementary Material. Further inquiries can be directed to the corresponding author.
