# Supplementary material for: GmHXK2 promotes the salt tolerance of soybean seedlings by mediating AsA synthesis, and auxin synthesis and distribution
Source: BMC Plant Biol. 2024 Jun 27;24:613. doi: 10.1186/s12870-024-05301-3 (PMC11210165; doi:10.1186/s12870-024-05301-3)
Supplement: Supplementary file 1 — Supplementary Material 1 [file 12870_2024_5301_MOESM1_ESM.docx]

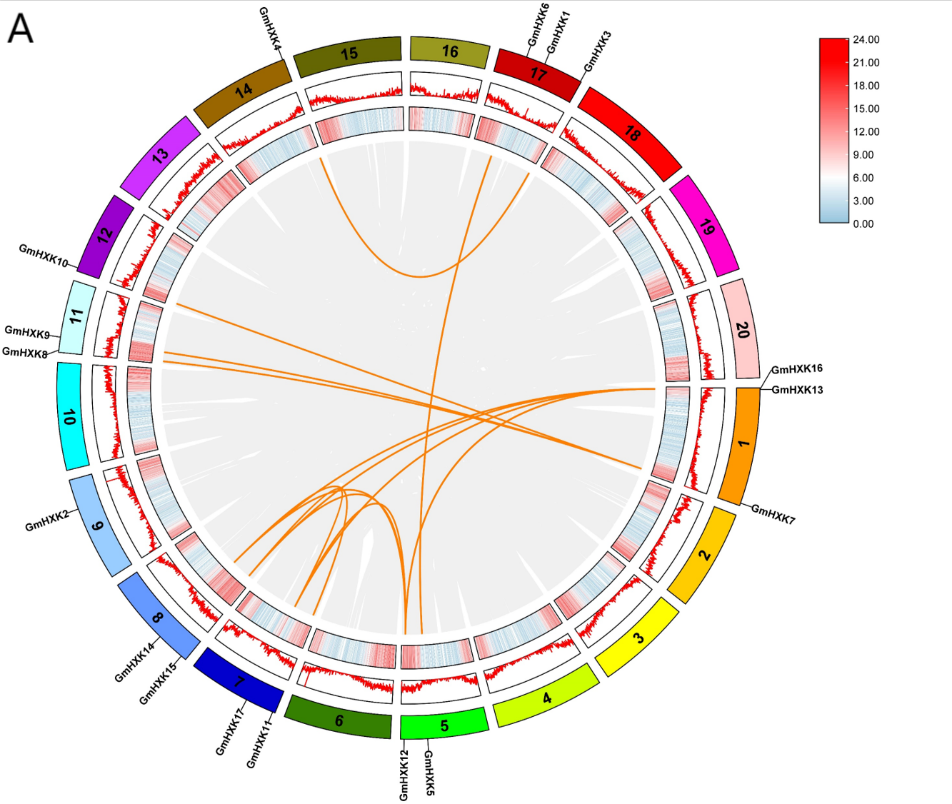

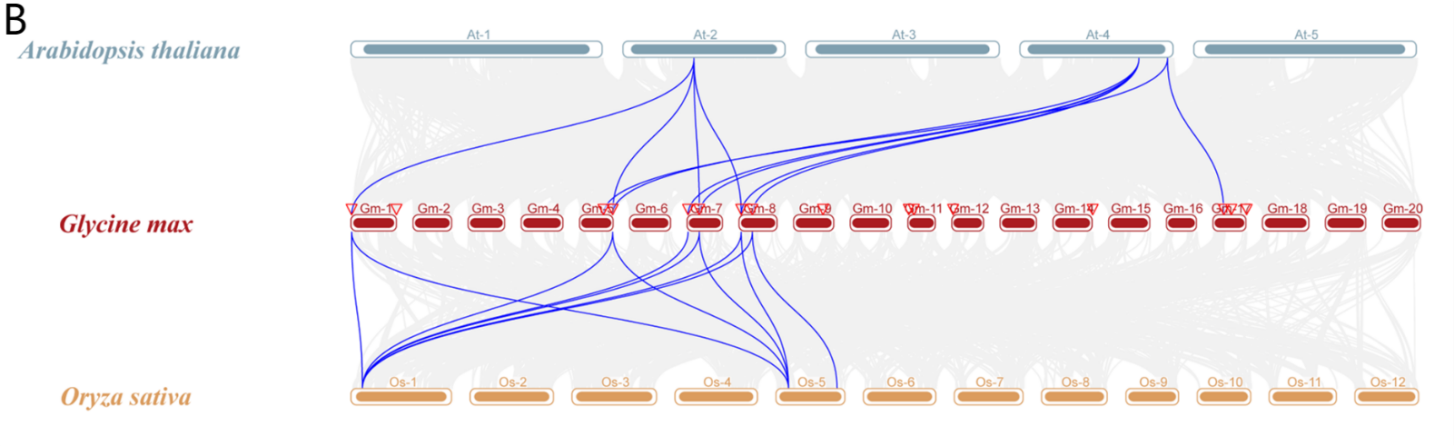


Fig. S1 (**A**) Collinearity analysis of hexokinase gene family in *Glycine max*. (**B**) Collinearity analysis of hexokinase gene family in *Glycine max*, *Arabidopsis thaliana* and *Oryza sativa*.
