# Supplementary figures and images for: GmHXK2 promotes the salt tolerance of soybean seedlings by mediating AsA synthesis, and auxin synthesis and distribution
Source: BMC Plant Biol. 2024 Jun 27;24:613. doi: 10.1186/s12870-024-05301-3 (PMC11210165; doi:10.1186/s12870-024-05301-3)

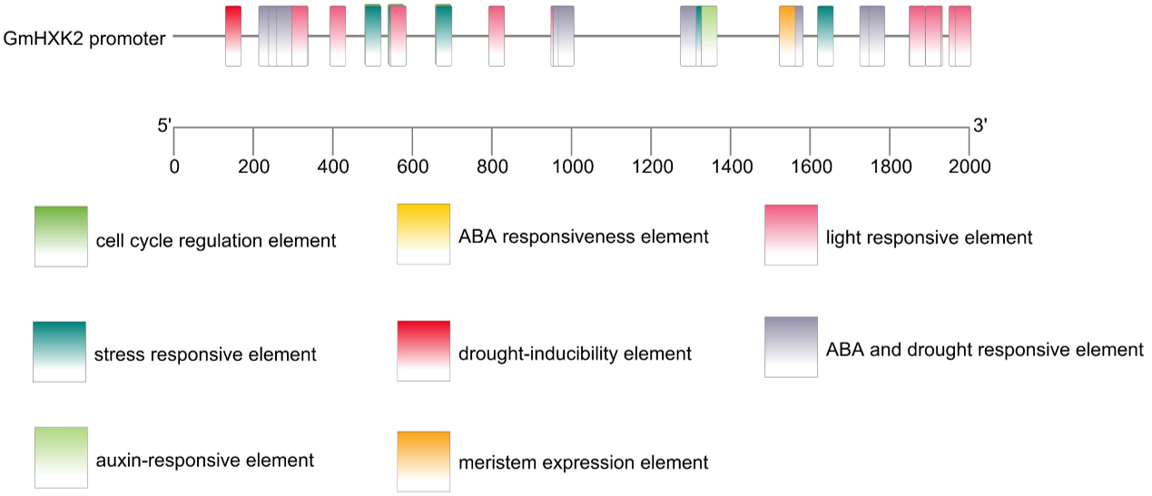


Fig. S2 Analysis of *GmHXK2* cis-regulatory elements

Supplement: Supplementary file 2 — Supplementary Material 2 [file 12870_2024_5301_MOESM2_ESM.docx]
